# Sexual and reproductive health of Syrian refugee women in Turkey: a scoping review within the framework of the MISP objectives

**DOI:** 10.1186/s12978-020-00948-1

**Published:** 2020-06-22

**Authors:** M. Çöl, N. Bilgili Aykut, A. N. Usturalı Mut, C. Koçak, S. U. Uzun, A. Akın, L. Say, L. Kobeissi

**Affiliations:** 1 Department of Public Health, Ankara University Faculty of Medicine, Ankara, Turkey; 2 Başkent University, Woman-Child Health and Family Planning Research and Implementation Center, Ankara, Turkey; 3 World Health Organization, Department of Reproductive Health and Research, Geneva, Switzerland

**Keywords:** Syrian refugee women, Sexual and reproductive health, Turkey

## Abstract

**Background:**

Turkey hosts the world’s largest community of Syrians displaced by the conflict. The Minimum Initial Service Package (MISP) is a coordinated set of priority reproductive health services. There is not any scoping review assessing the RH situation of Syrian refugees in Turkey within the framework of the MISP objectives. The objectives of this review is to identify the situation of sexual and reproductive health (SRH) among Syrian refugee women in Turkey, and document the health services provided for them in terms of the components of MISP. We hoped to show evidence of gaps and help guide future research to focus on priority areas to improve the range, quality, and access to SRH services and to recommend public health interventions.

**Method:**

The literature search was conducted in Turkish and English. Multiple electronic databases (Turkish Medline, Google Scholar, PubMed, Web of Science, Medline, Cochrane Database of Systematic Reviews, EBSCOHost, CINAHL, and Embase) were searched from January 2011 to May 2018. References published in the peer-reviewed literature, the grey-literature, and on websites were eligible for inclusion if they had conducted research on one or more of the following SRH topics specifically for Syrian women in Turkey: maternal and neonatal health/antenatal care, HIV and sexually transmitted infections, use of contraceptives, sexual violence, and services delivery and accessibility. References were excluded if any of the following criteria were relevant: not specific to Syrian women refugees in Turkey. Firstly, the titles and abstracts of the articles that were found were examined to determine if they met the eligibility criteria. Secondly, if the abstracts and titles met one or more of the eligibility criteria, the full text of the articles have been examined. Finally, standard forms were prepared and used to summarize the articles narratively. The results of the screening were recorded in Excel spreadsheets for comparison, and any disagreements among the researchers were resolved by consensus. The studies were grouped according to the MISP objectives.

**Results:**

A total of 24 publications were eligible for inclusion in the review. Consanguineous marriage rate was 56%. The rate of marriage under age 18 were very high. Mean age at first marriage was found to be between 18 and 20. The rate of antenatal care was inadequate. The rate of using a modern contraceptive method was 24% among married and all age groups of Syrian women. The rates of unmet family planning needs were about 35%. Among patients admitted to gynecology outpatient clinics, about half of the applicants were reported to have abnormal vaginal discharge. The reported rates of sexual violence were about 8%. Only 20% of Syrian women had regular gynecological visits.

**Conclusion:**

Overall, we conclude that early marriage, low modern contraceptive use, unmet need for contraception, sexual and gender-based violence are the major SRH issues reported. There is a need for further studies to identify the barriers limiting service uptake as well as to document successful practices. Long term strategies to improve the SRH status of Syrian refugee women should be developed with participation of all stakeholders. This review is significant in terms of that it is the first scoping review assessing the RH situation of Syrian refugees in Turkey within the framework of the MISP objectives. Based on the data of this review, relevant policy makers should consider to improve the SRH status of Syrian women refugees in Turkey.

## Plain ENGLISH summary

Turkey hosts the world’s largest community of Syrians displaced by the conflict. Refugee women are prone to exposure to a number of traumatic events and may face more barriers in accessing services. This review provides the situation of sexual and reproductive health (SRH) among Syrian refugee women in Turkey, and the health services provided for them.

Multiple electronic databases were searched from January 2011 to May 31, 2018. A total of 24 publications were eligible for inclusion in the review. Consanguineous marriage rate and the rate of marriage under age 18 were very high. Mean maternal age was lower, and adolescent pregnancy rates were higher compared to Turkish women. The rate of antenatal care was inadequate. The rate of using a modern contraceptive method was 24%. Among patients admitted to gynecology outpatient clinics, about half of the applicants were reported to have abnormal vaginal discharge. The reported rates of sexual violence were about 8%. Only 20% of Syrian women had regular gynecological visits. Although health services are made accessible free of charge to Syrian refugees in Turkey, early marriage, low modern contraceptive use, sexual and gender-based violence are among the major SRH issues reported. Long term strategies to improve the SRH status of Syrian refugee women should be developed with participation of all stakeholders.

## Introduction

The United Nations has declared the Syrian crisis one of the worst humanitarian crisis of the twenty-first century and a public health disaster [1]. Since March 15, 2011, the Syria conflict has resulted in an unprecedented level of population displacement, the majority of who have crossed borders to seek refuge in Turkey, Lebanon, Jordan, and Iraq [2, 3]. It is well established that refugees are likely to have worse health outcomes compared to local populations, as they are most vulnerable in terms of health status due to their difficult living conditions, difficulties in accessing services, health deteriorating after a difficult migration process, low income level, not knowing the language of the migrated country, not planning health systems for immigrants in the early period [4–6].

### Turkey’s response for refugees

Migration from Syria to Turkey started in April 2011 with 252 people, due to the open door policy the number of migrants increased steadily and reached nearly 3.7 million by August 2019 [2, 7].

Turkey has up to 63.4% of all Syrian refugees in the world according to UNHCR data [2, 7–9]. Turkey does not recognize Syrians as refugees but give them the “temporary sheltering status”, due to the geographic limitation of 1951-Geneva Convention. Migrants arriving from the only west of Turkey seeking international protection are subject to asylum procedures [10]. As the crisis continued, a series of legislation were put into effect, which allowed registered Syrians to receive basic health and social services [11–13].

It is estimated that 6% of Syrian refugees live in the camps established by the Turkish Government, and the rest are dispersed throughout the country in 2016 [14]. In Turkey, 46% of Syrian refugees are female and 51.2% of those females are in reproductive age (15–49 ages) [7].

Significant progress has been made to facilitate access of Syrian refugees to health services in Turkey [15]. The regulations have been started from the ten provinces where temporary shelter centers were located, by the time the right to access to free health services was expanded to cover all provinces [8, 16, 17]. Syrian doctors and nurses were trained on the functioning of the Turkish health care system and hired to work in migrant health centers run by Ministry of Health (MoH), to help to solve the language barrier [13].

Syrians in Turkey have access to health services at the migrant health centers as well as hospitals of the MoH. A total of 106 refugee health centers are providing services for Syrian refugees, and an additional 178 refugee health centers are planned to be established [18]. It should also be noted that health services for Syrians registered with the government are all offered health care free of charge [12]. It is not officially known how many of the Syrians in Turkey are not registered.

### Health problems of Syrian Refugee women

Women and children account for a disproportionate burden of morbidity among conflict-affected populations [19]. According to the UN Population Fund, globally 1.7 million Syrian women and girls need access to reproductive health (RH) services [20]. Globally, the limited availability of services, gender dynamics, limited awareness of where and how to access health services are all RH challenges [15]. It is reported in the literature that migrant women have problems of sexual violence, vulnerability to STIs associated with low risk perception and sexual abuse, higher risk of complications during pregnancy, increased risk of maternal death, higher risk of stillbirth and neonatal death, reduced access and use of healthcare services, lack of awareness of contraceptives methods and high unmet contraceptive need [21–25]. The focus on FP needs for refugees is critical. Because, both uptake of FP during refuge and displacement is often compromised and contraceptive methods can be difficult to reach in the countries that are migrated. As a result of unmet need for FP, unwanted pregnancies and negative consequences for the mother and child may occur [25].

Current data show that rates of early forced marriage, sexual violence, polygamy, consanguineous marriage, unwanted pregnancies, unsafe deliveries and maternal mortality among Syrian refugees are significantly higher when compared to Turkish women residing in Turkey [26–28].

The Minimum Initial Service Package (MISP), documented by the Inter-Agency Working Group on RH in 1996, is a coordinated set of priority RH services designed for the onset of an emergency to prevent excess morbidity and mortality [29]. Further, it supports the transition to comprehensive RH services as soon as possible. The MISP objectives are preventing excess maternal and newborn morbidity and mortality, prevention of HIV transmission and reducing the morbidity and mortality due to HIV and other sexually transmitted infections (STIs), planning for comprehensive RH services and access to these services and prevention and management of the consequences of gender based violence. The MISP entails the enforcement of the provision of priority activities such as the: provision of family planning (FP) services, syndromic treatment of STIs, prevention of mother to child transmission, ensuring availability of antiretroviral drugs, and distribution of culturally appropriate menstrual hygiene kits [29]. Providing these services is perceived as the minimum, yet extremely critical for ensuring refugee women’s sexual and reproductive health (SRH) needs are adequately met.

Despite the special efforts of Turkish government, MoH and NGOs in providing health services to the immigrants; assessment of the access to and utilization of reproductive health services and SRH outcomes is important due to the mobility of the Syrian population, the size of the needs related to RH services, the specific health risks of migrants, language/culture barriers, difficulties in accessing services, and the special needs of the women. Although reproductive health problems of migrants have been reported in the literature, there is still gaps about RH problems of Syrian refugees. There is not any scoping review assessing the RH situation of Syrian refugees in Turkey within the framework of the MISP objectives. Therefore, in this review we aim to provide an overview of the current RH situation of Syrian refugee women residing in Turkey to highlight the extent to which these services align with the MISP objectives. This review is hoped to show evidence of gaps and help guide future research to focus on priority areas to improve the range, quality, and access to RH services and to recommend public health interventions. The results of this review will guide policy makers in terms of solutions to improve the reproductive health of refugee women.

## Methods

This study is a mixed method scoping review conducted in accordance with the recommendations of PRISMA and MOOSE guidelines [30, 31].

### Data sources and search criteria

The MESH terms used for this search were: “Syrian”; “Refugee”; “Women”; “Adolescent Girls”; “Sexual Health”; “Reproductive Health”; “MISP” and “Turkey”. The search was conducted independently by three researchers, and all relevant identified studies were included in this scoping review.

The literature search was conducted in Turkish and English, using the following electronic databases and electronic collections for published studies meeting the eligibility criteria between January 2011 to May 2018: Turkish Medline, Google Scholar, PubMed, Web of Science, Medline, Cochrane Database of Systematic Reviews, EBSCOHost, CINAHL, and Embase. This was also coupled by a search using the grey literature, where we focused on relevant documents from: government, non-governmental organization (NGO) and health related websites. Proceedings from congress/conference books, reports and presentations on the SRH of Syrian refugee women in Turkey were also included. Reference lists of all research studies included in the review were also screened to identify additional studies for inclusion. With this broad and comprehensive search, we anticipate that this scoping review managed to access almost all relevant literature on the SRH of Syrian refugee women in Turkey.

### Eligibility criteria

References published in the peer-reviewed literature, the grey-literature, and on websites were eligible for inclusion if they had conducted research on one or more of the below SRH topics specifically for Syrian women in Turkey

- maternal and neonatal health/antenatal care (ANC),

-use of contraceptives,

- HIV and STIs,

- RH services delivery and accessibility, and

. -sexual violence.

#### References were excluded if any of the following criteria were relevant

- not specific to Syrian women refugees in Turkey.

The titles and abstracts of the articles that were found by means of the literature search were examined to determine if they met the eligibility criteria. If the abstracts and titles met one or more of the eligibility criteria, the full text of the articles have been examined. The evaluation process was same for the grey literature. Standard forms were prepared and used to summarize the articles narratively. The results of the screening were recorded in Excel spreadsheets for comparison, and any disagreements among the researchers were resolved by consensus. The studies were grouped according to the MISP objectives outline above. No meta- analysis was conducted in the context of the review, given the limited quality of survey designs and lack of for some of the SRH topics identified [32]. We also included relevant qualitative studies on the SRH of Syrian refugee women in Turkey. The study performed between March 2018 to May 2019.

## Results

**Fig. 1:**
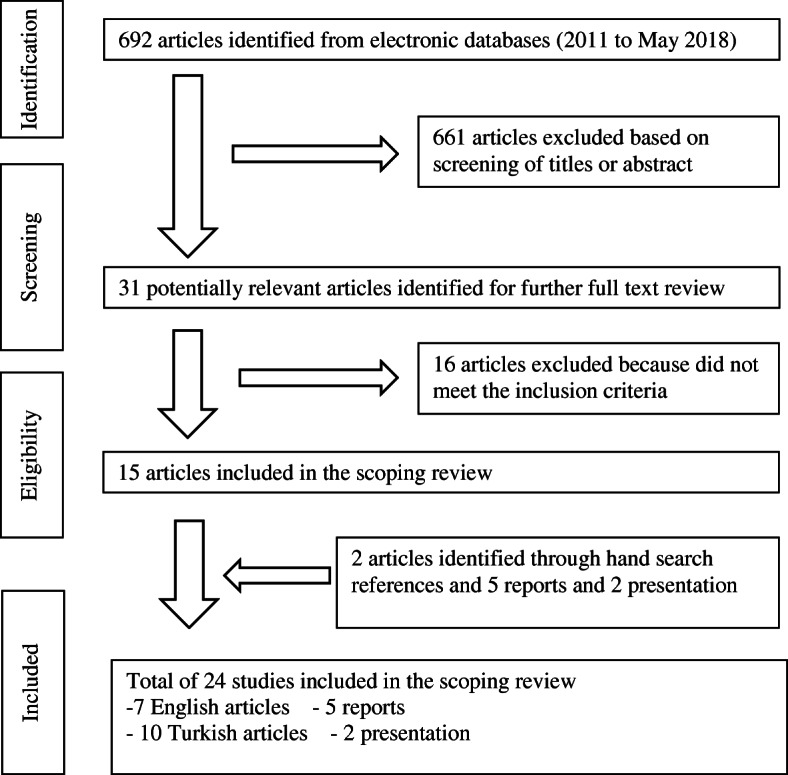
Studies and reports included in the scoping review

Initially, 692 articles were identified. Following title, abstract and full text screening of these articles, 15 articles met the inclusion criteria and were included in this review. Two additional articles identified based on the reference lists of the eligible included articles and five reports and two presentations from the grey literature were included based on the set eligibility criteria. Therefore, in total, 24 studies were found eligible and included in this scoping review. These articles were grouped into four main SRH domains in line with the MISP objectives (Fig. 1). The characteristics of all studies are presented in Table 1.

**Table 1:** Characteristics of included studies in the scoping review

Author(s)	Title	Publication Year	Journal	Study Design	Participants’ Characteristics
Erenel et al. [33]	Clinical characteristics and pregnancy outcomes of Syrian refugees: a case–control study in a tertiary care hospital in Istanbul, Turkey	2017	Arch Gynecol Obstet	Retrospective cohort study	300 Syrian-300 Turkish women who gave birth at a university hospital
Demirci et al. [34]	Birth characteristics of Syrian refugees and Turkish citizens in Turkey in 2015	2017	Int J Gynaecol Obstet	Retrospective study	545 Syrian-545 Turkish women who gave birth at a state hospital
Büyüktiryaki et al. [35]	Neonatal outcomes of Syrian refugees delivered in a tertiary hospital in Ankara, Turkey	2015	Conflict and Health	Descriptive study	457 Syrian who gave birth at a state hospital
Olgun [36]	Clinical characteristics of pregnant Syrian refugees and evaluation of birth results	2017	Dissertation	Retrospective study	584 Syrian-733 Turkish women who gave birth at a state hospital
**Şimşek et al.** [37]	The effect of health mediation model on access to basic health services among Syrian refugee women	**2017**	Book of 19. Turkey National Public Health Congress	Interventional study	64 Syrian refugee women
**Çift et al. **[38]	Comparison of Pregnancy Outcome and Serology Results in Turkish and Syrian Refugees Women	2017	Smyrna Medical Journal	Retrospective study	297 Syrian-300 Turkish women who gave birth at a state hospital
Bahadır [39]	Health status, healthcare access and factors influencing access to healthcare of the Syrian refugees living in a district setting in İzmir	2016	Dissertation	Cross-sectional study	257 Syrian refugee women living in İzmir
**Coşkun et al. **[40]	Fertility Awareness and Affecting Factors of Syrian Refugee Women	2015	Book of III. Intercultural Nursing Congress	Descriptive and qualitative study	200 Syrian refugee women who attended to a NGO center
**Karakaya et al. **[41]	Syrian refugee women’s fertility characteristics and influencing factors: a qualitative study	2017	The Journal of International Social Research	Descriptive and qualitative study	50 Syrian refugee women who attended to a NGO center
KAMER (Women’s Center Foundation) (NGO) [42]	The Report of Refugee Women in Five Cities	2017	KAMER Foundation Publications	Cross-sectional study	1138 Syrian refugee women living in 5 province located in southeast region of Turkey
Şimşek et al. [43]	A community-based survey on Syrian refugee women’s health and its predictors in Şanlıurfa, Turkey	2017	Women & Health	Cross-sectional study	458 Syrian women living in Şanlıurfa
Gümüş et al. [44]	Syrian refugee women’s reproductive health issues	2017	Journal of Women’s Health Nursing (KASHED)	Descriptive study	300 Syrian refugee women admitted to a university hospital
Torun et al. [45]	Health and health care access for Syrian refugees living in İstanbul	2018	International Journal of Public Health	Mixed method study	111 Syrian refugee women living in İstanbul
AFAD (Disaster and Emergency Management Authority) [46]	Syrian women in Turkey	2014	AFAD Publications	Cross-sectional study	3796 Syrian women living in the camps and 3552 Syrian women living outside camps
Yentür Doni et al. [47]	Investigation of the prevalence of trichomonas vaginalis among female Syrian refugees with the complaints of vaginitis aged between 15–49 years	2016	The Bulletin of Microbiology	Cross-sectional study	458 Syrian refugee women aged 15–49
Köse et al. [48]	Hepatitis A, B, C and HIV seroprevalence among Syrian refugee children admitted to outpatient clinics	2017	Le Infezioni in Medicina	Cross-sectional study	171 Syrian children (0–18 years) admitted to a state hospital
İnci et al. [49]	Analysis of HbsAg positivity rate before and after vaccination in Turkish and Syrian refugee pregnant women	2017	J Infect Dev Ctries	Retrospective study	2158 Syrian women and 2028 Turkish women
Keklik and Koruk [50]	Seroprevalence of hepatitis B and C in Syrian asylum seekers and their knowledge, attitude and risky behavior levels	2017	Book of 19. Turkey National Public Health Congress	Cross-sectional study	473 Syrian refugee living in Şanlıurfa
**Ördek **[51]	Syrians under “temporary protection” in Turkey and sex work.	2017	Red Umbrella Sexual Health and Human Rights Association (NGO)	Qualitative study and descriptive study	26 Syrian sex worker (22 women, 4 men)
Reyhanioğlu SÖ. UNFPA Turkey [52]	Reproductive Health and Family Planning Services in Syrian Asylum Seekers’ Humanitarian Assistance Program	2017	UNFPA	UNFPA Turkey Data	Syrian refugee women and girls in Turkey
Hacettepe University [53]	Strengthening the Accession of Syrian and Other Migrant Women to Sevices of Reproductive Health and Gender-Based Violence by Establishing Safe Areas and Women’s Health Advisory Centers for Women and Girls (in Turkish)	2016	Hacettepe University Women’s Research and Implementation Center (HUWRIC*/*HÜKSAM)	HUWRIC Project Data	Data of centers for Syrian refugee women
Özvarış ŞB [54]	Migration and Women’s Health	2017	Hacettepe University Women’s Research and Implementation Center (HUWRIC*/*HÜKSAM)	HUWRIC Data	Syrian refugee women admitted to HUWRİC centers
Turkish Medical Assocation [55]	War, migration and health	2016	Turkish Medical Association Publications	Review	–
MAZLUMDER (The Association for Human Rights and Solidarity for the Oppressed) (NGO) [56]	Report of Syrian Refugees Women Living outside the camp	2014	MAZLUMDER	Qualitative study	72 Syrian refugee women

### Maternal/new-born morbidity/mortality

20 articles on maternal/newborn morbidity/mortality were included. A cross-sectional study showed that 56% of Syrian women had consanguineous marriages [43]. Early and forced marriages are quite common, between 25 and 68% [39, 43, 44], mean age at first marriage was found to be between 18 and 20 [39, 40]. Adolescent pregnancy rate was between 10 and 30% [33, 36, 39, 40, 43, 44]. Three studies performed on hospital admission for delivery showed that mean maternal age of Syrian women was between 24 and 26 [33–35]. Of the Syrian women 36–73% received at least one ANC [33, 39, 40, 42, 43, 45]. Safe delivery rate was quite high. 91–99% of women had delivery with the help of trained health staff (doctors or midwives), for the 56–71% of the deliveries were performed by the attendance of a doctor [46], 74–90% of the deliveries were performed at hospital [39, 40, 45]. Cesarean delivery rates were between 9% - 39 [33–36, 38, 45]. Some studies showed that 23% of Syrian women had miscarriage [45], 11% had voluntary abortion [44]; mean number of abortion was between 1.6–1.7 lifelong [36, 40] mean number of induced abortion was 0.1 lifelong [34]. Some studies had information on neonatal health. According to these studies preterm birth rate was between 20 and 26% [35, 36]. Mean birth weight was between 3110 and 3136 g [34, 40]. An interventional study showed that health mediation model increased receiving ANC and decreased marriage at early ages among Syrian refugee women; and also improved other health measures such as vaccination rate, using any modern contraceptive method, and rate of breastfeeding [37]. In some qualitative studies, Syrian women stated that they considered pregnancy as a necessity, and they wanted to get pregnant especially for their husbands [40, 41].

It was observed that decision making around child bearing is led by the husband [40, 41] and the choice of son was generally an important value [43]. Unmet need for FP was reported as 36–38% [39, 43]. Use of any contraceptive method was between 20 and 63%, use of traditional method (withdrawal) is high and between 19 and 70% [40, 42, 43, 45]. The rate of using a modern contraceptive method was 24% among married and all age groups of Syrian women [39]. Use of intrauterine device (IUD) as most preferred modern method was between 16 and 34% [39, 40]. A qualitative study reported that awareness of modern contraceptive methods was high, but knowledge about the effectiveness was inadequate [41].

### STI and HIV prevention

Limited number of researches exist on STIs and HIV among Syrian refugee women in Turkey, and most of these studies were conducted on hospital admissions. Of Syrian refugee women living outside of the camps 51%, and of Syrian refugee women admitted to a university hospital 60% had smelly / abnormal vaginal discharge [43, 44]. 51% had STI symptoms [45]. 36% of those admitted to the hospital with vaginitis symptoms were diagnosed as trichomoniasis [47]. Anti-HIV seroprevalence rate was 2%, positive HBsAg rate was 4% and Anti-HCV positivity was 2% among Syrian refugee children admitted to outpatient clinics [48]. HBsAg seropositivity was found as 1.7 and 1.8% [49, 50]. Immunization rate against Hepatitis B was found as 7%, and awareness about Hepatitis B and C was low, only 2% of Syrian women had adequate information on transmission and prevention of hepatitis B and C [50]. A study conducted on Syrian sex workers showed none of them know about the centers offering anonymous HIV testing and counseling services in Turkey, and only 1/3rd of them use condoms regularly [51].

### Access to RH services

A total of 3 resources (2 presentations, 1 descriptive study) were included in the review. Studies on the access of Syrian women to the RH services were limited.

A total of 40 women’s health counseling centers, 4 youth centers, 20 social service centers and 5 key refugee service units were established by the support of UNFPA. These centers served 214.068 Syrian refugees in terms of female empowerment, prevention of gender-based violence, and response to violence [52, 53].

Utilization of some RH services was reported by one study. Only 20% of these women had regular gynecological visits, 10% had pap-smears, and 4% conducted breast self-examination [44].

### Gender based violence

Very few studies were found on gender-based violence among Syrian refugees in Turkey, primarily four documents were identified of which three were reports. These documents reported the harassment and sexual violence as 8–30% lifelong [42, 55, 56]. At a violence prevention center which was run by a university, 3 thousand women were provided services to prevent gender based violence and to heal victims. Nearly half of those were provided psychosocial support services [54].

## Discussion

We identified 24 studies to evaluate the SRH status of Syrian refugee women in Turkey and the health care services provided in line with the MISP objectives. According to the findings of this review, it was found that consanguineous marriages, early and forced marriages were quite common, safe delivery rate was quite high, frequency of at least one ANC visit was low, use of traditional method was high, the most used modern method was IUD, frequency of regular gynecological visits was low among Syrian refugee women in Turkey and nearly half of them had STI symptoms.

We found 13 indicators for MISP objective 1 (preventing excess maternal and newborn morbidity and mortality). These were frequency of early and forced marriage, adolescent pregnancy rate, frequency of at least one ANC, safe delivery rate, cesarean delivery rate, mean number of abortion, mean number of induced abortion, preterm birth rate, mean birth weight, frequency of use of any contraceptive method, use of traditional method, use of modern method, and unmet need for FP. We found 9 indicators for second objective of MISP (prevention of HIV transmission and reducing the morbidity and mortality due to HIV and other sexually transmitted infections (STIs). These were frequency of smelly / abnormal vaginal discharge, STI symptoms, Anti-HIV seroprevalence rate, HBsAg seropositivity, Anti-HCV positivity rate, immunization rate against Hepatitis B, frequency of awareness about Hepatitis B and C, frequency of awareness about the centers offering anonymous HIV testing and counseling services, and frequency of use condoms regularly. We found 2 indicators for MISP objective 3 (planning for comprehensive RH services and access to these services). These are frequency of regular gynecological visits and frequency of having pap-smear test. We found 3 indicators for MISP objective 4 (prevention and management of the consequences of gender based violence). These are frequency of the harassment and sexual violence, number of Syrian women provided services to prevent gender based violence, and number of Syrian women provided psychosocial support services.

There were huge discrepancies across the study designs of the eligible studies. Only one cross-sectional study [46] was identified that provided representative information on the SRH status of Syrian refugee women in Turkey, in 2014. Since then Syrian refugee population in the country almost tripled.

### Maternal/newborn morbidity and mortality

Maternal and infant mortality data among Syrian refugees in Turkey are lacking. Since no routine disaggregated data has been collected in this regard, the effect of the refuge on infant and maternal mortalities cannot be accurately assessed in Turkey. Disaggregated health service records should be routinely collected and updated by data from the field, coupled with regular evaluation and dissemination with the health and scientific society. Maternal and child mortality data, particularly, should be regularly monitored and evaluated. Further, periodic population-based health surveys should be carried out [46, 57].

Before the crisis, home deliveries were quite common in Syria (45%) [58]. However, delivery rate by health care providers for Syrian refugees was found quite high in Turkey (over 90%) [46] as in Jordan (%100) and Lebanon (99%) [59]. On the other hand, the indicated studies reported the rates of ANC defined as at least one antenatal care during pregnancy not as high [33, 39, 40, 42, 43, 45]. However ANC rate were increasing in Syria before crisis (71 to 88% between 2002 and 2009) and after the crisis decreased to 77% [58, 60]. In Lebanon and Jordan, ANC rates of Syrian refugees were high and paralleled the rates with pre-war Syrian rates [59]. These findings suggest insufficient access to health services in Turkey after migration. Indicated studies reported various barriers to ANC services, language being the most cited barrier. Also, social isolation and lack of awareness of health care services and how to access them were among the other important cited factors [61–65]. None of the included studies reported or evaluated the quality of ANC received as well as the utilization of postnatal care among Syrian women refugees in Turkey.

Child marriages and adolescent pregnancies are important risk factors for maternal and newborn health (mortality and morbidity). The review showed that child marriages and adolescent pregnancies were quite common among Syrian refugee women in Turkey [33, 36, 39, 41, 44]. Rates of child marriages were higher than those reported for Turkish women (15%), as well as when compared to the reported rates in Syria (13%) before the crisis, according to UNICEF data [66]. Although, early marriages could be considered generally as part of traditional practices, these high rates could be associated with the perceived need of social protection for girls against socio-economic hardships and violence (including sexual violence) [67, 68]. Comprehensive SRH programming focused on raising awareness on the negative consequences of child marriages and adolescent pregnancies are necessary.

In Turkey, pregnancies can be legally terminated until the end of 10th week of, in case of medical necessity, it is legal to terminate it after the 10th week [69]. The review reported that induced abortion rates among Syrian refugees were similar to those reported among the Turkish population [34, 44]. Literature on this, however, remains very limited the impact of migration on abortion rates should be further examined in detail.

Newborn health is also equally affected by displacement and refuge [34–36, 63, 64]. The preterm birth rates were similar to the rates reported among Syrian refugees in Lebanon (%26) [59] and were significantly higher to the rates reported among Turkish women (11%) [36].

According to UNICEF data total fertility rate in Syria was 3 in 2012, at the beginning of the crisis, while it was 2 in Turkey for the same year [70]. Especially in rural areas in the northern and southern regions of Syria, fertility rate was higher. It should be noted that the majority of Syrian refugees who were displaced into Turkey were primarily from the northern parts of Syria, where fertility rates were higher [41]. Some of the studies identified for this review showed that high fertility trend of Syrian women continued after migrating to Turkey [39–41, 43]. Further, these studies showed that husbands are the main decision makers on child bearing [40, 41]. Preference for sons, husbands’ polygamy, also put pressure on Syrian women to give more birth [8, 41, 43].

In general, uptake of FP during refuge and displacement is often compromised [70, 71]. Studies identified this review showed that the use of modern contraceptive methods was low, and the most used modern method was IUD [39, 40, 43]. The health system in Turkey has fairly a well-established infrastructure for FP. As a result of this, the rate of using any modern contraceptive method is 47% in Turkey [72]. The focus on FP needs for refugees is critical. Although, sufficient infrastructure is available for the provision of contraceptive services to Syrian refugee women in Turkey, the uptake and use of these services remain limited. Understanding the challenges and the barriers impacting services’ uptake such as language, socio-cultural factors and traditional beliefs is essential to improve FP awareness, uptake of services as well to limit the implications of unwanted pregnancies.

### STI and HIV prevention

As a result of increased violence and insecurities associated with displacement and conflict, refugees are often faced with an increased risk for engagement in risky sexual behavior and thus at higher risk of contracting STIs [21, 22, 73]. This further challenged by the insufficient provision of RH services to prevent and manage STIs appropriately [25].

Studies identified for this review provided very limited data on the status of STIs and HIV prevention among Syrian refugees in Turkey. The fact that most of these studies were conducted at the health facilities has significant implications on the generalizability of these findings. Having multiple partners by women is not generally common practice in Arab societies [74]. Hence, the high reported STIs prevalence can be attributed to sexual violence and/or survival sex [75]. According to one study conducted among Syrian sex workers in Turkey, the rates of condom use was low and the knowledge about where to access HIV counseling and testing was equally poor [51]. Further, adequate awareness and prevention of HIV transmission from mother to infant should also be taken into consideration. The findings of the limited number of studies, on the awareness and burden of STIs suggest the need to conduct more research, stronger epidemiological response and service delivery tailored to the needs of Syrian refugees.

### Access to RH services

The studies in this review were able to demonstrate a broad range of challenges and barriers impacting access and use of SRH services among Syrian refugees in Turkey. While refugee women are at higher risks of unwanted pregnancies, unsafe abortions and deliveries, maternal deaths, gender based violence and sexual abuse, they have limited access to basic health services such as FP, ANC, safe delivery. The results of this review converge with these findings, in spite of the strengths associated with the health system in Turkey [39].

In Turkey, during the immigration from Syria, provision of health care services to the refugees and also access to these services has drastically improved [13, 76]. Syrian refugees in Turkey can have access to health services both in hospitals of MoH and migrant health centers. All therapeutic and preventive health services provided to Turkish citizens by MoH are provided free of charge to Syrian refugees (8, 16, 17). It should be noted that the registered Syrian refugees in Turkey enjoy the same health care services benefits as those of the Turkish citizens [77]. However, the high mobility of refugees between provinces, fears from registration, the hopes to seek refuge to other European countries as well as language, are all important factors impacting the Syrian refugees’ access to health services [44, 61]. Satisfaction rates for health services were high [76, 78]. However, no satisfaction data were available on satisfaction with the access and provision of SRH services among Syrian refugee women.

It is known that during the early onset of the Syrian refugee crisis in Turkey, capacity issues, especially the Arabic speaking personnel or interpreters, posed a significant challenge and required a rapid response [71]. Consequently, in the subsequent years, the MoH established dedicated refugee health centers. Health professionals among the Syrian refugees were hired to work in these centers, following training on the health system in Turkey and certification. Trainings were conducted by the MoH in collaboration with World Health Organization, several NGOs, universities and UNFPA [18].

### Gender based violence

It is estimated that approximately 6000 women were raped in Syria since the surge of the crisis until 2015 [79]. This rate is further aggravated by the high rates of sexual assaults, harassments, forced marriages, and polygamy, among Syrian refugee women.

As indicated above, very few studies investigated the prevalence of sexual violence among Syrians refugees in Turkey. Findings from these studies estimated lower rates of sexual violence than global data [44, 56]. In traditional communities, women who are exposed to sexual violence may avoid seeking help because of fears of stigmatization and/or prosecution. It is extremely important to identify women with these special needs during registration. Counseling services should be provided to women who have been exposed to every type of gender based violence and the security and privacy of women should be safe-guarded and protected, when these services are provided [55, 80]. Evidence informed research (addressing multiple dimensions and perspectives from legal, cultural, educational, psychosocial as well as medical) is also recommended to drive forward protective support measures and mechanisms to prevent sexual violence and adequately address and manage STIs including HIV among this vulnerable population [15].

This review is limited to the published literature in Turkish and English that were found in the databases assessed. It is possible that a publication bias may have occurred; however, to compensate of that, unpublished reports and presentations were as well explored and included if they met the search eligibility criteria to the search. Also, the study design of many of the accessed studies had relatively small sample sizes and for few SRH topics such as violence, there were very few studies conducted.

## Conclusion

This review is significant in terms of that it is the first scoping review assessing the RH situation of Syrian refugees in Turkey within the framework of the MISP objectives. Child marriages, adolescent pregnancies, consanguineous marriages, inadequate ANC, high unmet needs for modern contraception, low awareness of the risk of STIs, prevention and management including HIV, inadequate regular gynecological visits and sexual violence constitute important SRH challenges faced by Syrian women refugees in Turkey. More research is needed on the barriers to SRH access as well as drivers of maternal and infant mortality among this population. Despite the provision of the health care services at free of charge, the utilization of these services among Syrian refugees in Turkey remain below the desired levels. The MISP is a set of priority activities and services that are recognized as standard care for RH in emergency situations. Information and data on the needs and challenges of the Syrian population in Turkey is rapidly becoming outdated and requires regular monitoring. More implementation research is needed to identify barriers and challenges as well as inform evidence-based strategies to improve Syrian women refugees’ access to SRH services. These recommendations for relevant health authorities, NGOs and international organizations could be considered to improve the SRH status of Syrian women refugees in Turkey.

## Data Availability

The data that support the findings of this study are available from the corresponding author upon reasonable request.

## Abbreviations

AFAD: Disaster and Emergency Management Authority
ANC: Antenatal Care
FP: Family Planning
IUD: Intrauterine Device
MISP: Minimum Initial Service Package
MoH: Ministry of Health
NGO: Non-Governmental Organization
RH: Reproductive Health
SRH: Sexual and Reproductive Health
STIs: Sexually Transmitted Infections
UNFPA: United Nations Population Fund
UNICEF: United Nations International Children’s Emergency Fund
